# Circulating tumor microemboli (CTM) and vimentin+ circulating tumor cells (CTCs) detected by a size-based platform predict worse prognosis in advanced colorectal cancer patients during chemotherapy

**DOI:** 10.1186/s12935-016-0373-7

**Published:** 2017-01-05

**Authors:** Dejun Zhang, Lei Zhao, Pengfei Zhou, Hong Ma, Fang Huang, Min Jin, Xiaomeng Dai, Xiumei Zheng, Shaoyi Huang, Tao Zhang

**Affiliations:** 1 Cancer Center, Union Hospital, Tongji Medical College, Huazhong University of Science and Technology, Wuhan, 430022 Hubei People’s Republic of China; 2 Wuhan YZY Medical Science & Technology Co., Ltd., Wuhan, 430075 Hubei People’s Republic of China

**Keywords:** Circulating tumor cells, Circulating tumor microemboli, Colorectal cancer, Survival, Vimentin

## Abstract

**Background:**

Circulating tumor cells (CTCs) detected in peripheral blood (PB) of cancer patients can be identified as isolated CTCs and circulating tumor microemboli (CTM). This study aimed to evaluate the prognostic value of CTM detection and CTC phenotype in advanced colorectal cancer (CRC) patients during chemotherapy.

**Methods:**

A size-based platform for CTC isolation was applied. PB samples (5 ml) from 98 advanced CRC patients during 2–6 cycles chemotherapy were collected for CTC detection, and CTC count was correlated to patient’s clinicopathological characteristics and clinical outcome. And CTC phenotype was measured by immunofluorescent staining and evaluate the predictive significance on survival in 32 CTCs-positive patients with advanced CRC.

**Results:**

Forty-eight of 98 patients were CTCs-positive, including 18 CTM-positive patients, and CTC detection was positively correlated with lymphatic invasion (*P* = 0.049), TNM stage (*P* = 0.023), and serum CEA level (*P* = 0.014). Moreover, Kaplan–Meier survival and Cox regression analyses revealed that the presence of CTCs was an independent factor for poor PFS and OS (*P* < 0.05) in advanced CRC patients during chemotherapy, and CTM-positive patients had shooter survival than isolated CTCs-positive patients (*P* < 0.05). Furthermore, patients with vimentin+ isolated CTCs/CTM had shorter PFS and OS compared with CK+ CTCs (*P* < 0.05).

**Conclusions:**

This study provided evidence that the presence of CTCs was positively correlated with poor prognosis, and furthermore, CTM and vimentin+ CTCs predicted poorer survival, which indicated that CTM and vimentin+ CTCs detected by a sensitive platform could be used to improve prognostic value of CTCs in advanced CRC patients under treatment.

## Background

Colorectal cancer (CRC) is the third most common cancer in male and the second most common in female worldwide, and contributes the fourth cause of cancer death in male and the third in female [1]. For advanced CRC patients, although many patients benefit from chemotherapy to some extent, for some patients excessive chemotherapy was unnecessary due to inefficiency, moreover, multiple adverse effects seriously lower their life quality [2]. Therefore, new prognostic factors which could be used to identify patients who would benefit from chemotherapy are needed.

Circulating tumor cells (CTCs) non-invasively isolated from peripheral blood can serve as a “liquid biopsy” and as a source of valuable tumor markers. Many studies reported that CTC detection had prognostic and therapeutic significance in CRC [3–7]. Moreover, in advanced CRC patients, the presence of CTCs before and during treatment had been proved to be an independent predictor of progression-free survival (PFS) and overall survival (OS) [3, 6], and a key factor to improve the accuracy in assessing the effectiveness of first-line treatment [7].

However, CTC detection, enumeration and molecular characterization are quite challenging, because CTCs are rare in peripheral blood of patients. The Veridex CellSearch system (Veridex LLC, Raritan, NJ) utilizes magnetic beads coated by anti-EpCAM antibody to capture cells followed by the fluorescence staining to identify CTCs, defined as CK8/18/19+/DAPI+/CD45− cells [8]. However, EpCAM expression is dependent on the local microenvironment and is down-regulated in disseminated cells [9]. Epithelial-mesenchymal transition (EMT) of tumor cells is induced in the bloodstream [10], which leads to mesenchymal tumor cells with stem-like phenotype [11, 12], and loss of epithelial phenotype [13]. This is quite probably the reason why the CTC detection rates and counts in the CellSearch system are generally low. For example, 17 of 66 non-metastatic CRC patients (26%) had ≥2 CTCs per 7.5 ml peripheral blood [14], and in another study, only 19 of 239 preoperative CRC patients (~8%) had ≥1 CTC per 7.5 ml peripheral blood [15]. Therefore, CTCs as an independent prognostic marker, need a more sensitive method to further facilitate the evaluation of CTC detection.

Here, a sensitive size-based platform for CTC isolation was applied, which could filter the hemocytes with small diameter and capture the tumor cells with relatively big diameter, followed by Romanowsky dye and immunofluorescent staining to identify CTCs. In this study, peripheral blood samples (5 ml) from 98 advanced CRC patients during 2–6 cycles chemotherapy were collected to detect CTCs for Romanowsky dye staining, then CTC levels were correlated with clinicopathological characteristics and patient’s survival. Moreover, CTC phenotype was measured by immunofluorescent staining in 32 CTCs-positive patients with advanced CRC. It was demonstrated that CTC detection by a size-based platform was positively correlated with lymphatic invasion, TNM stage, serum CEA level and poor survival, and CTM and vimentin+ CTCs predicted poorer survival in advanced CRC under treatment.

## Methods

### Patients

Ninety-eight patients with advanced CRC during 2–6 cycles chemotherapy were recruited in Cancer Center, Union Hospital, Huazhong university of science and technology, from January, 2013 to April, 2013, and peripheral blood samples from patients were collected. The TNM classification of CRC was based on American Joint Committee on Cancer (AJCC) 7th edition. The clinicopathologic characteristics of patients were classified according to the chart records, as showed in Table 1.

**Table 1:** Relationship between circulating tumor cells (CTCs) and clinicopathological characteristics in advanced colorectal cancer

Characteristics	No. of patients (%)	CTCs		*P* value
		Positive	Negative	
All patients	98 (100)	48 (49.0)	50 (51.0)	
*Gender*				
Male	61 (62.2)	31 (50.8)	30 (49.2)	0.640
Female	37 (37.8)	17 (45.9)	20 (54.1)	
*Age (median 52, years)*				
<60	60 (61.2)	30 (50.0)	30 (50.0)	0.800
≥60	38 (38.8)	18 (47.4)	20 (52.6)	
*Tumor size (cm)*				
<5	43 (43.9)	20 (46.5)	23 (53.5)	0.666
≥5	55 (56.1)	28 (50.9)	27 (49.1)	
*Tumor location*				
Colon	58 (59.2)	29 (50.0)	29 (50.0)	0.808
Rectum	40 (40.8)	19 (47.5)	21 (52.5)	
*Histology differentiation*				
Poor	23 (23.5)	18 (78.3)	5 (21.7)	0.043*
Middle	54 (55.1)	23 (42.6)	31 (57.4)	
Well	21 (21.4)	7 (33.3)	14 (66.7)	
*Depth of invasion*				
T1 + T2	15 (15.3)	6 (40.0)	9 (60.0)	0.135
T3	25 (25.5)	11 (44.0)	14 (56.0)	
T4a	47 (48.0)	22 (46.8)	25 (53.2)	
T4b	11 (11.2)	9 (81.8)	2 (18.2)	
*Lymphatic invasion*				
N0	30 (31.3)	12 (38.7)	19 (61.3)	0.049*
N1	22 (22.2)	7 (31.8)	15 (68.2)	
N2a	22 (22.2)	13 (59.1)	9 (40.9)	
N2b	24 (24.2)	16 (66.7)	8 (33.3)	
*TNM stage*				
III	17 (17.3)	4 (23.5)	13 (76.5)	0.023*
IVa	22 (22.5)	9 (40.9)	13 (59.1)	
IVb	59 (60.2)	35 (59.3)	24 (40.7)	
*CEA (ng/ml)*				
≤10	54 (55.1)	20 (37.0)	34 (63.0)	0.014*
>10	44 (44.9)	28 (63.8)	16 (36.4)	
*CA199 (U/ml)*				
≤37	57 (58.2)	24 (42.1)	33 (57.9)	0.151
>37	41 (41.8)	24 (58.5)	17 (41.5)	

Italic values indicate statistically significant associations

* *P* ≤ 0.05

This prospective study was double-blinded in terms of blood draw, CTC detection and identification. For the purpose of this study, healthy donors were those without abnormal cells detected by this size-based platform for CTC isolation in peripheral blood.

The informed consent approved by ethics committee of Union Hospital, Huazhong university of science and technology had been obtained from all patients before examination. All procedures performed in studies involving human participants were in accordance with the ethical standards of the ethics committee of Union Hospital, Huazhong University of science and technology and with the Helsinki declaration and its later amendments or comparable ethical standards.

### CTC detection by a size-based platform

The 5 ml blood sample of advanced CRC patient was diluted up to 8 ml with 0.9% physiological saline containing 0.2% paraformaldehyde, then measured on an automated testing platform following manufacturer’s instructions, as described in an earlier study by Vona et al. [16]. This platform was composed of a membrane with 8 μm size pores and a automated testing device. The captured cells including abnormal cells and residual haemocytes on the membrane were stained with Romanowsky dye (eosin and methylene blue) and immunofluorescent staining. The candidate CTCs were identified independently by 3 senior cytopathologists.

### Immunofluorescent staining

The captured tumor cells on the membrane were processed with Cytofix/Cytoperm Fixation/Permeabilization solution (BD, New Jersey, USA) for 10–15 min, incubated with 10% Goat Serum (Jackson, West Grove, USA) for 30 min at room temperature, then incubated with anti-CK8/18/19, anti-vimentin (Abcam Trading (Shanghai) Company Ltd., Shanghai, China) and anti-CD45 (Santa, Texas, USA) antibody overnight at 4 °C. The next day they were incubated with secondary antibodies, Alexa Fluor 488-conjugated goat anti-mouse, Alexa Fluor 546-conjugated goat anti-rabbit, Cy5-conjugated goat anti-rabbit (InvitrogenTM, Thermo Fisher Scientific, Waltham, USA), and Hoechst (SIGMA, St. Louis, MO) for 1 h at room temperature. Then they were imaged by fluorescence microscope.

### Statistical analysis

All data were analyzed using SPSS 16.0 statistic software (SPSS Inc., Chicago, IL, USA). The associations between CTCs and clinicopathologic variables were evaluated with χ^2^ tests. Survival curves were calculated using the Kaplan–Meier method. Factors of prognostic significance were investigated with the univariate and multivariate Cox regression model. For all tests, the *P* ≤ 0.05 indicated statistical significance.

## Results

### Abnormal cells detected by a size-based platform for CTC isolation in peripheral blood of patients with advanced CRC

**Fig. 1:**
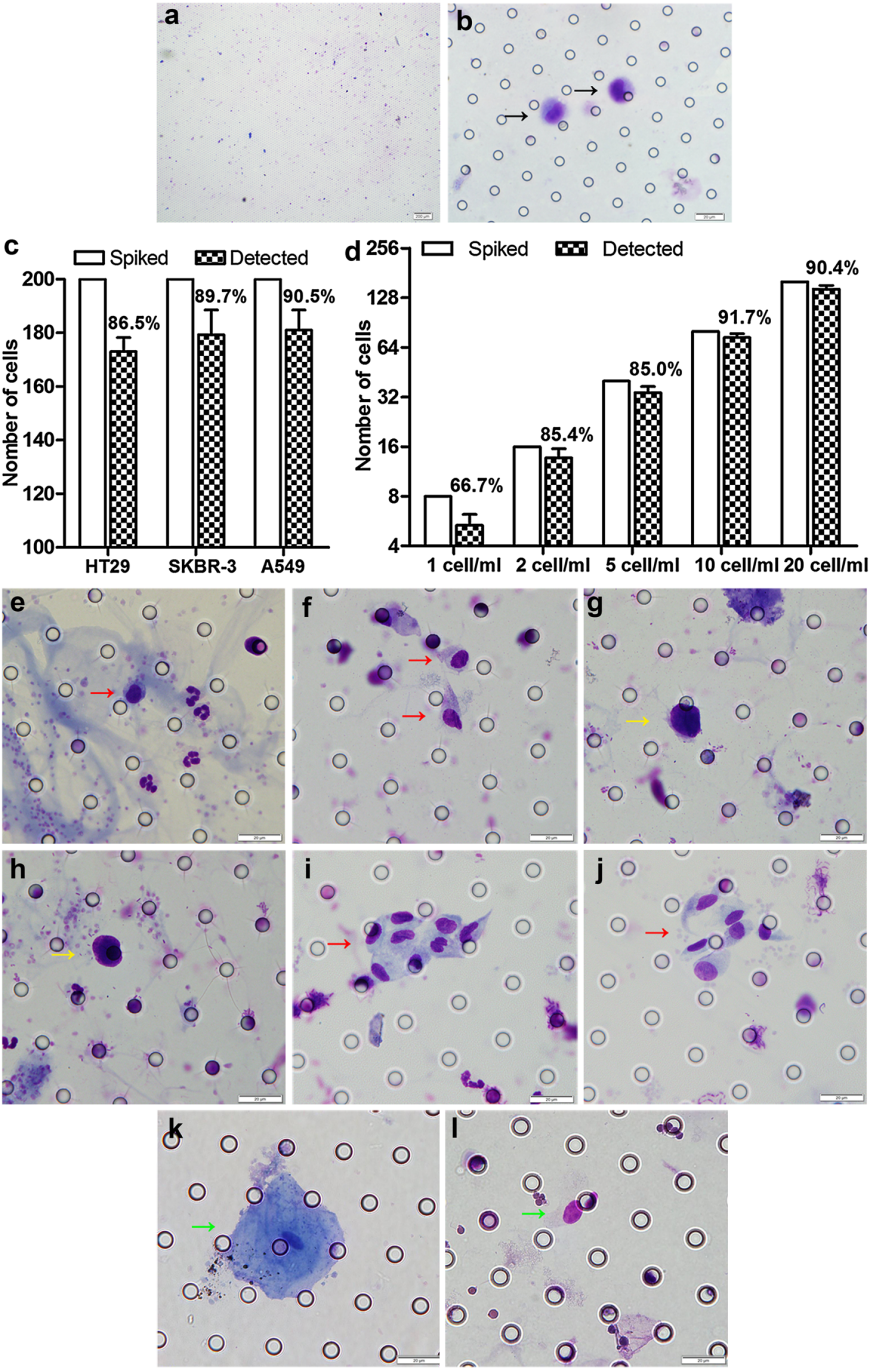
Abnormal cells detected in peripheral blood (PB) of advanced CRC patients. **a** The clear background of a membrane in the filter after Romanowsky staining. **b** The spiking HT29 cells captured by the size-based platform for CTC isolation (as indicated by the *black arrows*). **c** The capture efficiency of cancer cell linces HT29, SKBR-3 and A549. **d** The sensitivity of isolating HT29 cells. **e**, **f** The single CTC (as indicated by the *red arrows*) detected in PB. **g**, **h** The suspected CTC (as indicated by the *yellow arrows*) in PB. **i**, **j** CTM (as indicated by the *red arrows*) detected in PB. **k** Epithelial cells (as indicated by the *green arrows*) detected in PB. **l** Endothelial cells (as indicated by the *green arrows*) detected in PB (**a**, ×10 magnification; **b**, **c**, ×60 magnification; **f**–**m**, ×100 magnification)

In this study a size-based platform for CTC isolation was applied. This platform was mainly composed of a filter membrane with 8 μm size pores and an automated testing device. A spiking test was conducted to test the capture efficiency and sensitivity of this platform, in which HT29 colorectal cancer cells were added into 5 ml peripheral blood of healthy donors. the transparent membrane in the filter got a clear background after CTC isolation and Romanowsky staining, which facilitated the procedure of indentifying CTCs and CTC phenotype (Fig. 1a, b). The results showed that this method for isolating CTCs was reliable and robust (Fig. 1c, d).

Based on the criteria proposed by other researchers [16–18] and our own experience, there were 6 criteria of cell morphological characteristics for evaluating abnormal cells captured in peripheral blood: (1) the nuclear atypia: irregularity of nuclear shape, may be nodular or lobulated etc.; (2) a high nuclear–cytoplasmic ratio: >0.8; (3) a large cell diameter (the long diameter): >15 μm; (4) the hyperchromatic nuclei were dyed unevenly (due to the increase of chromatin and the thicker particles in cancer cells, the nucleus was hyperchromatic); (5) the thickened nuclear membrane was sunken, wrinkled and jagged; (6) the nuclear chromatin margination (nucleus side-shift), or a large nucleoli, or abnormal nuclear division.

Abnormal cells captured by this method were identified as CTCs in colorectal cancer, only if they met no less than 4 criteria above, or met the 6th criterion and any other 2 criteria (Fig. 1e, f). If they met any 3 criteria except the 6th criterion, or met only the 6th criterion, they were identified as the suspected CTCs (Fig. 1g, h). Besides, CTC cluster composed of three or more CTCs was recognized as circulating tumor microemboli (CTM) (Fig. 1i, j), while other cell clusters were recognized as the suspected CTM. However, some cells should not be present in peripheral blood normally (e.g. epithelial cells, endothelial cells) (Fig. 1k, l), or were of undetermined origin, all those cells were regarded as non-blood cells.

### The relationship between CTCs/CTM and clinicopathological characteristics in advanced CRC with treatment

**Fig. 2:**
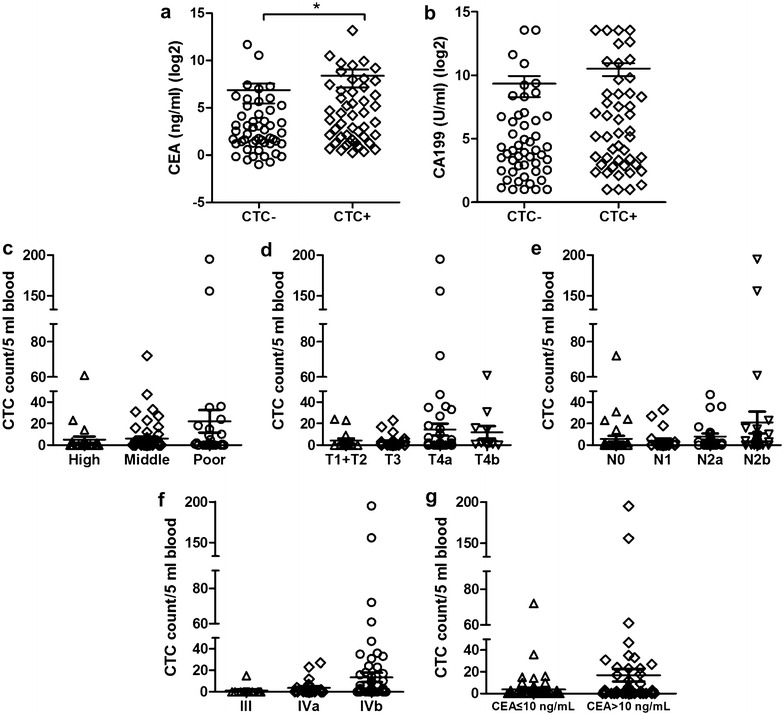
The relationship between CTCs/CTM and clinicopathological characteristics in advanced CRC. **a** Serum CEA levels in CTC-positive patients were higher than CTC-negative patients (*P* = 0.0155). **b** There was no statistical significance in serum CA199 levels between CTC-positive and CTC-negative patients (*P* = 0.0887). **c** The correlation of CTC count with tumor de-differentiation (poor vs. middle, *P* = 0.0191; poor vs. high, *P* = 0.0359). **d** CTC count of patients with depth of invasion (T4a vs. T4b, *P* = 0.7826; T4a vs. T3, *P* = 0.3708; T4a vs. T1 + T2, *P* = 0.4762). **e** The correlation of CTC count with lymphatic invasion (N2b vs. N0, *P* = 0.0429; N2b vs. N1, *P* = 0.0361; N2b vs. N2a, *P* = 0.1037). **f** The correlation of CTC count with TNM stage (IVb vs. III, *P* = 0.0186; IVb vs. IVa, *P* = 0.1019). **g** CTC count of patients with CEA > 10 pg/ml was more than CEA ≤ 10 pg/ml (*P* = 0.0026)

In this study, ninety-eight advanced CRC patients during 2–6 cycles chemotherapy were subjected to CTC isolation and enumeration, forty-eight patients were CTCs-positive, including 18 CTM-positive patients. The association of CTCs with the clinicopathological variables of patients was shown in Table 1. CTCs were positively correlated with tumor de-differentiation (*P* = 0.004), lymphatic invasion (*P* = 0.049), TNM stage (*P* = 0.023), and serum CEA level (*P* = 0.014). By contrast, no significant association was found between CTCs-positive and other clinicopathological characteristics (*P* > 0.05 for all others), such as gender, age, tumor size, tumor location, serum CA199 level, and depth of invasion (Table 1). Serum CEA levels in CTCs-positive patients were higher than CTCs-negative patients (334.8 ± 194.7 vs. 115.6 ± 71.43 ng/ml, *P* = 0.0155) (Fig. 2a), while there was no statistical significance in serum CA199 levels between CTCs-positive and CTCs-negative patients (1486 ± 498.7 vs. 651.1 ± 339.2 U/ml, *P* = 0.0887) (Fig. 2b).

Furthermore, CTC enumeration of all 98 advanced CRC patients ranged from 0 to 195 (mean ± SE: 9.663 ± 2.775), and CTM enumeration ranged from 0 to 17. And CTC enumeration was increasing with decreased tumor de-differentiation (poor vs. middle, *P* = 0.0191; poor vs. high, *P* = 0.0359), increased lymphatic invasion (N2b vs. N0, *P* = 0.0429; N2b vs. N1, *P* = 0.0361; N2b vs. N2a, *P* = 0.1037), TNM stage (IVb vs. III, *P* = 0.0186; IVb vs. IVa, *P* = 0.1019) and serum CEA level (CEA > 10 vs. CEA ≤ 10 ng/ml, *P* = 0.0026) (Fig. 2c–g).

### CTCs/CTM predicted poor survival in advanced CRC patients under treatment

**Fig. 3:**
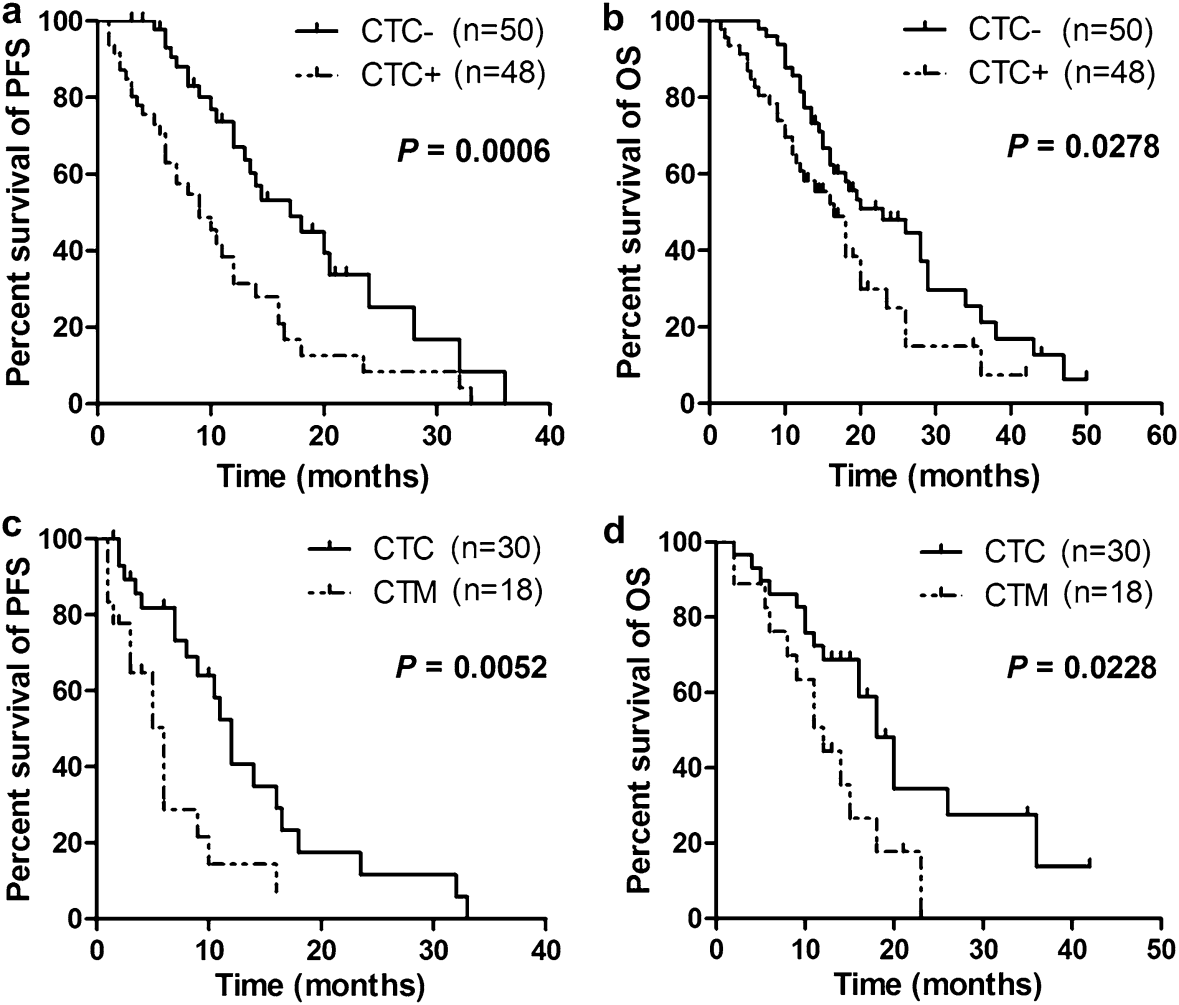
The relationship between CTCs/CTM and PFS/OS in advanced CRC. **a**, **b** The PFS and OS of CTC-positive patients were shorter than CTC-negative patients (*P* = 0.0006, *P* = 0.0278). **c**, **d** The PFS and OS of CTC-positive patients were worse than CTM-positive patients (*P* = 0.0052, *P* = 0.0228)

Based on univariate Cox regression analyses for all factors (Table 2), CTCs (*P* < 0.0001), lymphatic invasion (*P* = 0.042), TNM stage (*P* < 0.001), and high CEA level (*P* = 0.0027) were closely related with PFS. The multivariate Cox regression model further demonstrated that CTCs (*P* = 0.015) and TNM stage (*P* = 0.013) were independent prognostic factors for shorter PFS (Table 2). And the Kaplan–Meier survival curves showed that CTCs-positive patients with advanced CRC had a significantly unfavorable PFS (9 vs. 17 months, *P* = 0.0006) (Fig. 3a), and furthermore, CTM-positive patients had shorter PFS than CTCs-positive patients (6 vs. 12 months, *P* = 0.0052) (Fig. 3c).

**Table 2:** Univariate and multivariate analysis of prognostic factors for progression-free survival (PFS) and overall survival (OS) in advanced colorectal cancer

	PFS				OS			
	HR	95% CI		*P*	HR	95% CI		*P*
Univariate analysis								
*Gender*								
Male vs. Female	1.242	0.746	2.070	0.404	0.907	0.540	1.523	0.711
*Age*								
<60 vs. ≥60	0.959	0.572	1.608	0.875	0.977	0.593	1.612	0.929
*Tumor size*								
<5 vs. ≥5	1.173	0.708	1.944	0.535	0.766	0.465	1.259	0.293
*Location*								
Colon vs. Rectum	0.945	0.564	1.584	0.831	1.331	0.809	2.190	0.261
*Differentiation*								
Well vs. Middle vs. Poor	0.746	0.521	1.068	0.109	1.000	0.696	1.437	0.999
*T*								
T1+T2 vs. T3 vs. T4a vs. T4b	1.164	.871	1.555	0.304	1.081	.810	1.442	0.597
*N*								
N0 vs. N1 vs. N2a vs. N2b	1.255	1.009	1.562	0.042*	1.507	1.210	1.875	<0.001***
*TNM*								
II+III vs. IVa vs. IVb	2.027	1.383	2.971	<0.001***	1.552	1.091	2.207	0.015*
*CEA* *(ng/ml)*								
≤10 vs. >10	1.828	1.070	3.121	0.027*	1.452	0.884	2.385	0.141
*CA199* *(U/ml)*								
≤37 vs. >37	1.620	0.973	2.698	0.064	1.123	0.684	1.842	0.647
*CTCs*								
Negative vs. Positive	2.870	1.716	4.801	<0.0001***	1.664	1.003	2.761	0.048*
Multivariate analysis								
*N*								
N0 vs. N1 vs. N2a vs. N2b	1.169	0.930	1.469	0.180	1.499	1.198	1.876	<0.001***
*TNM*								
II + III vs. IVa vs. IVb	1.687	1.115	2.553	0.013*	1.580	1.086	2.298	0.017*
*CEA* *(ng/ml)*								
≤10 vs. >10	1.258	0.712	2.224	0.429				
*CTCs*								
Negative vs. Positive	1.993	1.141	3.483	0.015*	1.148	0.679	1.943	0.606

Italic values indicate statistically significant associations

* *P* < 0.05, *** *P* < 0.001

Moreover, based on univariate Cox regression analyses for all factors (Table 2), CTCs (*P* = 0.048), lymphatic invasion (*P* < 0.001), and TNM stage (*P* = 0.015) were closely related with poor OS. Although the multivariate Cox regression model demonstrated that lymphatic invasion (*P* < 0.001) and TNM stage (*P* = 0.017) were independent prognostic factors for PFS but not CTCs (Table 2), the Kaplan–Meier survival curves showed that CTCs-positive patients with advanced CRC had a significantly unfavorable OS (16.5 vs. 23 months, *P* = 0.0278) (Fig. 3b), and CTM-positive patients had worse OS than CTCs-positive patients (12 vs. 18 months, *P* = 0.0228) (Fig. 3d).

### Vimentin+ isolated CTCs/CTM predicted worse survival in advanced CRC patients under treatment

**Fig. 4:**
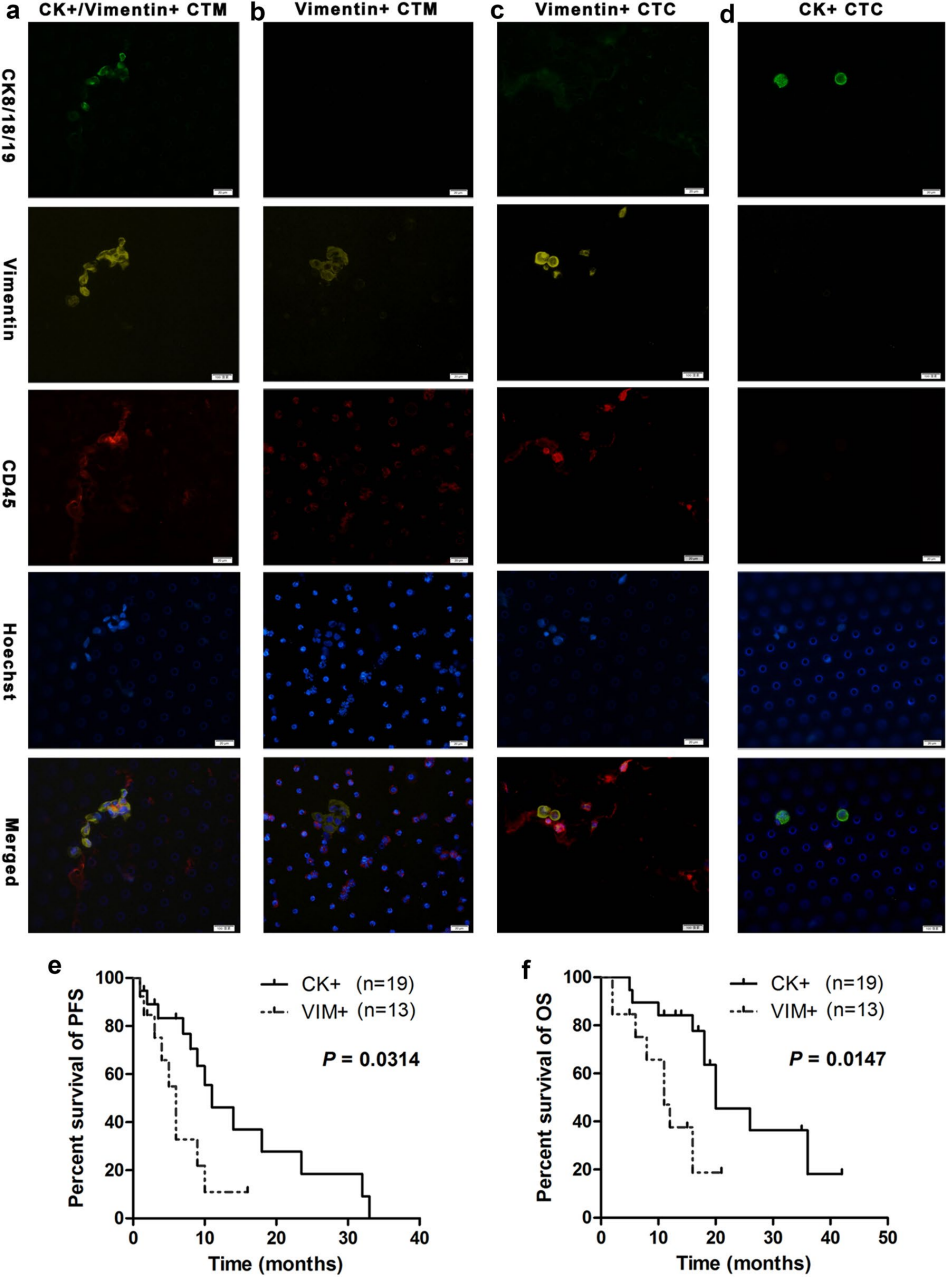
The relationship between vimentin+ CTCs and PFS/OS in advanced CRC. The captured tumor cells were stained with anti-CK8/18/19 antibody for epithelial marker (*green fluorescence*), anti-vimentin antibody for mesenchymal marker (*yellow fluorescence*), anti-CD45 antibody for leukocytes (*red fluorescence*), and hoechst for nucleus (*blue fluorescence*). The CTM detected in peripheral blood of patients were CK+/Vimentin+/CD45− (**a**) or CK−/Vimentin+/CD45− (**b**) phenotype. The isolated CTCs were CK−/Vimentin+/CD45− (**c**) and CK+/Vimentin−/CD45− (**d**) phenotype. **e**, **f** Patients with vimentin+ CTCs had worse PFS/OS compared with CK+ CTCs (*P* = 0.0314, *P* = 0.0147)

Thirty-two CTCs-positive patients were subjected to CTC isolation again to identify CTC phenotype by immunofluorescence. The samples were stained with anti-CK8/18/19 antibody (epithelial marker), anti-vimentin antibody (mesenchymal marker), anti-CD45 antibody (for leukocytes), and hoechst (for nucleus). In this study, four CTC phenotypes were detected: CK+/Vimentin+/CD45− CTM (Fig. 4a), CK−/Vimentin+/CD45− CTM (Fig. 4b), CK−/Vimentin+/CD45− isolated CTCs (Fig. 4c), and CK+/Vimentin−/CD45− isolated CTCs (Fig. 4d). For further analysis, 13 patients with vimentin+ CTCs/CTM (CK+/Vimentin+/CD45− CTM, CK−/Vimentin+/CD45− CTM, CK−/Vimentin+/CD45− isolated CTCs) and 19 patients with CK+ CTCs (CK+/Vimentin−/CD45− isolated CTCs) were identified. Interesting, it was found that all of CTM (detected in 11 of 11 patients) were vimentin-positive, while most of the isolated CTCs (detected in 19 of 21 patients) were CK-positive. Moreover, the Kaplan–Meier survival curves showed that advanced CRC patients with vimentin+ CTCs had significantly shorter PFS and OS compared with CK+ CTCs (6 vs. 11 months, *P* = 0.0314; 11 vs. 20 months, *P* = 0.0147) (Fig. 4e, f).

## Discussion

CTC detection in peripheral blood was recognized as “liquid biopsy” in solid tumors, because it could be performed easily, frequently, and less invasively [19, 20]. There was increasing evidence which prove CTCs as the clinical marker for diagnostic, prognostic, and pharmacologic purposes [21, 22]. Hence, CTC detection and characterization had become a research focus worldwide.

Although many studies about CTCs proved that high baseline CTC count was positively correlated with worse prognosis in colorectal cancer by CellSearch system [6, 23, 24], the CTC detection rate and count in CellSearch system were generally low, and many approaches of CTC isolation had been developed recently. In this study, we applied a size-based platform for CTC isolation, and the spiking tests showed the capture efficiency and sensitivity of this platform was reliable and robust. Moreover, the CTC detection rate in advanced CRC patients during 2 ~ 6 cycles chemotherapy was 49% (48 of 98 patients), which was significantly higher than that detected by CellSearch system (data showed in meta-analysis) [23, 24], and it was consistent with the results of another study which compared CTC detection rate of the size-based platform and the CellSearch system in esophageal carcinoma [25]. The high sensitivity of this size-based platform could be mainly attributed to two factors: Firstly, the CellSearch system only regarded tumor cells with epithelial phenotype in peripheral blood as CTCs, which did not take other properties and processes which were associated with malignant potential into consideration, such as EMT, cohesive and collective cell migration [22]. Secondly, this size-based platform captured malignant cells by the difference of diameter and deformability between abnormal cells and haemocytes, hence it could isolate more abnormal cells for further identifying CTCs. However, when comparing the CTC detection rates by ISET (isolation by size of epithelial tumor cells) in some studies [26–29], there was a subtle difference in this study. The discrepancy might due to the heterogeneity of different cancers, different stages of tumor, and whether undergoing treatment or not, etc.

We also observed the relationship between CTCs and clinicopathological characteristics, as shown in Table 1. It was found that CTCs were associated with tumor de-differentiation, lymphatic invasion, TNM stage, and serum CEA level, which were consistent with the results of previous studies [30, 31]. In addition, serum CEA values in CTCs-positive patients were higher than CTCs-negative patients, which indicated that patients with high CEA levels had more opportunities to be CTCs-positive. Moreover, CTC count was increasing with decreasing tumor de-differentiation, increasing lymphatic invasion, TNM stage, and serum CEA level. Therefore, although the decisions on stage of disease still did not include the results of CTC assessment, the presence of CTCs might be an adjunct to staging [32], and it could be expected that CTC detection predicted the properties and processes of the disease (e.g. lymphatic invasion, TNM stage, and serum CEA level).

This study found that the presence of CTCs was associated with decreased survival in advanced CRC patients with 2–6 cycles chemotherapy, and Cox regression analyses showed that CTC detection was an independent prognostic factor for survival, which was consistent with previous studies [23, 24, 33, 34]. Notably, it was reported that the relationship between CTC detection and prognosis was more significant and convincing when the blood samples were collected during treatment than at baseline [23, 24], which indicated that sample collection during treatment was preferable for CTC detection to predict CRC patient’s outcomes. That was the reason why we recruited the advanced CRC patients with 2–6 cycles chemotherapy in this study. Moreover, CTM was captured by this size-based platform, and CTM-positive patients with advanced CRC had worse survival than isolated CTCs-positive patients. It was reported that tumor cells within CTM could be protected from anoikis and were relatively resistant to cytotoxic drugs [35], and CTM was an independent prognostic factor [35, 36]. Hence, CTM would be more malignant and aggressive than isolated CTCs.

CTCs were comprised of heterogeneous cells including epithelial tumor cells, tumor cells undergoing EMT and tumor stem cells etc. [12, 37, 38], and circulating epithelial tumor cells had been shown to respond to therapy in the same way as the primary tumor [39], while the detection of EMT markers (LOXL3 and ZEB2) for CTCs in mCRC predicted poor survival and therapy response during treatment [40], hence CTC molecular characterization could offer the potential to better understand the biology of metastasis and resistance to established therapies [19]. In this study CTC phenotype was measured by immunofluorescent staining for CK8/18/19 (epithelial marker) and vimentin (mesenchymal marker), and it was found that all CTM were vimentin-positive, while most of the isolated CTCs were CK-positive. Moreover, patients with vimentin+ CTCs had worse survival than CK+ CTCs. To our knowledge, this was the first study that evaluated the prognostic role of CTCs with epithelial and mesenchymal phenotype in advanced CRC patients during treatment.

## Conclusion

In this study, it was found that the presence of CTCs was associated with decreased survival, and was an independent prognostic factor for outcome in advanced CRC patients during chemotherapy. Moreover, patients with CTM had shorter survival than those with isolated CTCs, and patients with vimentin+ CTCs had worse survival compared to those with CK+ CTCs. Therefore, this study had demonstrated that CTM and vimentin+ CTCs could be used to improve prognostic value of CTCs in advanced CRC patients under treatment.

## Abbreviations

AJCC: American joint committee on cancer staging
CTCs: circulating tumor cells
CTM: circulating tumor microemboli
CA125: carbohydrate antigen 125
CEA: carcinoembryonic antigen
CK: cytokeratin
CRC: colorectal cancer
EMT: epithelial to mesenchymal transition
OS: overall survival
PFS: progression-free survival
PB: peripheral blood
TNM: tumor-node-metastasis
